# Professor John Rarity

**DOI:** 10.1038/s41377-025-02113-4

**Published:** 2026-01-03

**Authors:** Yining Zhang

**Affiliations:** The LSA Editorial Office in Edinburgh, The Light Publishing Group, Edinburgh, United Kingdom

**Keywords:** Optics and photonics, Physics

From demonstrating the first “path entanglement” experiments in the 1980s to enabling secure quantum communication links stretching from mountaintops to space, Professor John G. Rarity has been a central figure in translating quantum mechanics from a subject of philosophical debate to a driver of real-world technology.

Currently Professor of Optical Communication Systems at the University of Bristol, and a key member of its Quantum Engineering Technology Labs (QET Labs), Rarity’s work bridges the most fundamental tests of quantum physics with engineering solutions that make them practical, reliable, and deployable. His achievements include the first violation of Bell’s Inequality over 4 km of optical fibre (1994), the landmark 144 km free-space quantum key distribution (QKD) link between La Palma and Tenerife (2007), and recent efforts to miniaturise QKD payloads for CubeSats and even handheld consumer devices.

In 2023, these contributions were recognised with the Micius Quantum Prize—one of the highest international honours in quantum information science—awarded jointly with Professor Nicolas Gisin for their pioneering work on quantum communication.

We spoke with Professor Rarity about the evolution of his career, the technological breakthroughs that have shaped the field, and his vision for a secure quantum network spanning the globe.

**Q1. When did your interest in physics first begin, and what led you towards quantum optics specifically?**

I was very much the kind of student who was encouraged along by parents and teachers. I was bright and naturally good at science, maths, and chemistry, but I didn’t have a clear sense of direction. Their advice was always, “You should just do physics.” At the time, I imagined myself doing something more adventurous, but physics was the subject that came easiest to me, so I followed that path—not out of passion initially, but because it seemed like the natural choice.

The turning point came during my MSc (in Biophysics and Bioengineering), when I began real research. Up until then, I passed exams without difficulty, though I wasn’t at the very top because of other side interests. But once I had a project of my own in the lab, everything changed. I discovered the thrill of solving problems independently and the satisfaction of seeing results that belonged to me. I didn’t look forward to lectures, but I looked forward to working in the lab every day.

My MSc project involved light scattering with photomultipliers to study electrophoresis and cell motion. Early on, I learned that photon-counting methods were far superior to analogue detection for these experiments. That insight set me on a course towards photon counting and photon statistics. For my PhD at the Royal Military College of Science, I studied light scattering from colloids, moving gradually from bioengineering into physical chemistry. My first postdoc, in the early 1980s, was in a leading light-scattering group that had pioneered photon statistics. By then, I had gained a strong grounding in how light is quantised and why counting photons offered such a clean, sensitive method compared to conventional approaches.

Around this time, in the lab next door, colleagues inspired by Len Mandel were exploring nonlinear crystals that produced photon pairs. I found that work fascinating and took over the experiments when the lead researcher moved on. I was joined by an excellent collaborator Paul Tapster and we began trying to control light at the single-photon level—anti-bunching photons, regulating their number with shutters, and even creating light below the usual shot-noise limits.

In 1987, Rodney Loudon pointed us towards something even more surprising: the peculiar behaviour of two photons meeting at a beam splitter, what is now called the Hong–Ou–Mandel effect. The idea that two independent photons could “leave holding hands,” always exiting together rather than separately, was astonishing. When we first heard about it, we rushed into the lab to reproduce it. We succeeded, but by the time we checked the journals, Hong, Ou, and Mandel had already published their landmark experiment. Even so, that moment was transformative. We realised we were operating at the very edge of discovery, competing with the best in the world. That gave us the drive and confidence to push further.

So, while physics didn’t start as a passion for me, the excitement of research—especially the counterintuitive beauty of quantum optics—captured me completely. That’s how I found my way into this field.

**Q2. How did your earlier research experiences shape your scientific approach afterwards?**

My earliest experiences, particularly with dynamic light scattering and colloids, were actually a bit frustrating. To get meaningful results, you had to perform many experiments, rely heavily on statistical analysis, and often depend on someone else to prepare perfect samples. It was rarely clear what the outcome would be until the very end. That lack of predictability made it hard to feel a sense of direct progress.

What I loved about quantum optics was the contrast. Here, the theory was strong and predictive. You could work something out on paper, then go into the lab expecting a specific result—and more often than not, you saw exactly what the equations had foretold. That alignment between theory and experiment was deeply satisfying.

Of course, there were still surprises. For example, we once sent photon pairs into a Mach–Zehnder interferometer. A visiting theorist from Malvin Teich’s group came by, and his predictions were close, but not quite right. We observed two-photon interference fringes—oscillations at twice the frequency you’d expect—because the two photons together carried the coherence of the pump beam. This effect extended far beyond the individual coherence lengths of the photons themselves. It was counterintuitive, and it forced us to refine the theory. Those were the moments when the lab taught us something new, and they were tremendously exciting.

It was during this period that we also recognised the link between the Hong–Ou–Mandel effect and entanglement. That led us to develop path-entanglement experiments that, for a time, pushed us ahead of groups like Mandel’s. The thrill came from seeing our work at the forefront—making it into Physical Review Letters several times between 1987 and 1994. That sense of pushing the boundaries was addictive.

So in short, my early experiences taught me two things: first, the frustration of experiments that depended too heavily on others, and second, the joy of physics where theory and experiment danced closely together. That shaped my preference for work where I could predict, test, and then be surprised in ways that advanced the theory itself.

**Q3. As your career progressed, when did you first realise that quantum effects could be harnessed for practical technologies?**

There were several moments, but one stands out clearly. In the late 1980s, we began reading about unusual schemes for secure communications—using quantum mechanics to share secrets. The idea was intriguing, but I didn’t fully grasp the potential until I met Artur Ekert at a NATO conference in Cortina d’Ampezzo in 1991, which, rather memorably, was held at a ski resort. We like to joke that entanglement based quantum key distribution was invented on the ski slopes.

At the time, though, my focus was still on testing the foundations of quantum mechanics—verifying that entanglement really did produce the “spooky action at a distance” predicted by theory, and that experiments would rule out classical alternatives. It was only after long discussions with Artur—sketching on posters and whiteboards—that the practical application clicked for me.

The realisation was simple but profound: in all of our experiments, a photon leaving one port of an interferometer could be labelled as a “0,” and if it left the other port, as a “1.” A photon cannot split and appear in both places—it must be one or the other. Suddenly, we saw how entangled photons could generate shared random keys, with perfect correlations between distant parties.

We already had in our lab the components for such an experiment—long-distance interferometers capable of sending photons far apart while maintaining entanglement. From that point, I was convinced: single photons could be used not just to probe quantum theory, but to build entirely new forms of secure communication.

This was in 1992, when Artur and I published our first scheme showing how his theoretical ideas could be realised with interferometers rather than polarisation. We later demonstrated it in the lab. Looking back, it was an extraordinary moment of transition—when a strange and counterintuitive effect became the seed of a practical technology.

**Q4. Your late 1980s demonstration of path entanglement is often cited as an experimental milestone. How did you approach this challenge with the technology at the time?**

To begin with, we certainly didn’t set out thinking we were going to “invent” path entanglement. At the time, we weren’t even using that language. What we were really asking was: in a standard interferometer, a single photon can take two paths, recombine, and interfere with itself. But what happens if you send two photons through an interferometer and then recombine them—do they interfere as well?

In some sense, that was exactly what we had already been doing with the Hong–Ou–Mandel experiment: sending two photons into an interferometer-like setup, recombining them at a beam splitter, and looking for unusual correlations. When we extended this work, we discovered something new. Because of quirks in the nonlinear crystals we used, the output photons came in a small rainbow of frequencies. Each photon could be blue- or red-shifted, but their total energy always had to add up to that of the pump.

We realised we could exploit this by opening the apertures wide, producing broadband photons. That led to interference patterns shaped like a sinc function, which is the Fourier transform of a top-hat distribution. Then, by blocking the central part of the spectrum, we created two sharp peaks and observed beating between them.

At first, we thought we were seeing “two-colour photons”—each blue *and* red—beating together. But after careful thought, we recognised that wasn’t quite right. What we actually had were photons with strong correlations: if a blue-shifted photon appeared on one side, its partner on the other side had to be red-shifted, and vice versa. That was the real breakthrough. We could reinterpret the setup as a kind of two-slit interferometer for each colour, with the red and blue photons linked by their shared paths.

By relabelling it this way, we began to see the first hints of path entanglement. If the red photon took a particular route, its blue partner was constrained to follow a correlated path. Yet until we made a measurement, neither photon could be said to have taken one route or the other—they were in a superposition of possibilities. By adjusting the interferometer phases, we demonstrated that no definite path could be assigned. Measuring one photon revealed the path information of the other, and that was the essence of entanglement.

Effectively, we had created a two-path interferometer for pairs of photons. Other groups later simplified the concept—for example, using Mach–Zehnder interferometers—and Franson’s proposal of a Bell inequality test became a practical tool for exploring these ideas. Our experiments, though, showed that entanglement could live *between* the paths themselves.

This line of work also led, somewhat inadvertently, to the interferometer scheme later cited in the Micius Prize. That approach uses long and short paths in two interferometers, connected by long fibres, to create time-bin entanglement. When the photons are recombined, some arrive together and interfere, enabling quantum key distribution (QKD). Today, many QKD systems rely on this kind of path or time-bin encoding.

At the time, of course, our main motivation wasn’t building technology—it was probing entanglement in new ways. It was only later, especially in discussions with Artur Ekert, that we realised these path states could be digitised. By the mid-1990s, people were beginning to talk about “qubits,” and it became natural to say: a qubit can live in one path, the other path, or a superposition of both. That connection to Bell inequalities and quantum information came a little later, but our early experiments laid much of the groundwork.

**Q5. In 1994, your team achieved a violation of Bell’s Inequality over 4** **km of optical fibre. What technical advances made this possible, and what did it mean for the community?**

Our earlier experiments with path entanglement were fascinating, but they weren’t practical for long-distance transmission. To extend entanglement over kilometres of optical fibre, we needed a simpler and more robust approach.

In the 1994 experiment, we used two nearly identical photons and sent them through unbalanced interferometers placed several kilometres apart. Each interferometer gave the photon two possible routes: a short path or a long path. When we examined the detection events, there were four possibilities: short–short, long–long, short–long, and long–short. The mixed cases—short–long and long–short—didn’t interfere, so we filtered them out. What remained were the long–long and short–short cases, and those two possibilities interfered beautifully.

By isolating those interference peaks in our coincidence counts, we could directly test Bell’s inequality. The correlations we measured violated the classical limit, demonstrating quantum entanglement across 4 km of fibre. That was a major step forward.

But there was more. If you interpret the outputs of each interferometer as binary outcomes—detector one as “1,” detector two as “0”—you suddenly see the connection to quantum key distribution. When the phases of the two interferometers are aligned, a detection event on one side always corresponds to the same outcome on the other. If I get a “1,” my partner also gets a “1.” If I get a “0,” so do they. By adjusting the phases, you can flip the correlation so that a “1” here corresponds to a “0” there.

The result is perfect correlations between two distant parties, generated by entangled photons travelling through ordinary telecom fibre. Neither photon has a definite path until measurement, because the outcome arises from the interference of the long–long and short–short possibilities.

For the community, this experiment was significant for two reasons. First, it showed that entanglement could survive transmission over real-world distances, proving that quantum effects were not confined to tabletop setups. Second, it pointed directly toward practical applications. What had been a purely foundational test of nonlocality was also, quite clearly, the basis for a fibre-based QKD system.

That dual impact—simultaneously advancing our understanding of quantum theory and laying the groundwork for technology—was why the 4 km Bell test became such a milestone.

**Q6. How did these early results influence the trajectory of quantum key distribution research?**

Our discussions with Artur Ekert were pivotal. Together, we proposed an interferometer-based scheme for entanglement-based key distribution. One of the key insights was that, while polarisation-based approaches were elegant in theory, they were problematic in practice when transmitted through optical fibres. Fibre polarisation tends to drift unpredictably, and at that time, classical telecom systems weren’t designed to preserve polarisation—they relied on intensity modulation, simple on–off keying, or polarisation-insensitive formats.

So, we asked: why not use interferometers instead? By sending photons through unbalanced interferometers, we could encode information in their relative phases rather than in polarisation. The setup was conceptually simple: an incoming photon is split, takes both paths, and then recombines. When two such systems are linked, the resulting correlations provide the raw material for quantum key distribution.

This was essentially the fibre-optic version of QKD. By interpreting the interferometer outputs as “0”s and “1”s, we could apply the same principles as in polarisation-based schemes but without the instability issues. Depending on the phase settings, the correlations could be aligned (1–1 and 0–0) or anti-aligned (1–0 and 0–1). Either way, the security came directly from quantum mechanics: the measurement outcomes were perfectly correlated, yet inherently unpredictable.

That insight helped direct the field. By the mid-1990s, many groups working on QKD began building interferometer-based systems. In parallel, others pursued free-space schemes using polarisation, but in fibre, interferometry became the dominant approach.

For us, it was more than just a technical detail—it meant our single-photon experiments had a practical purpose. That, in turn, opened the door to new funding opportunities, since agencies could now see a path from foundational science to secure communications. Personally, it also suited me: I wanted the freedom to pursue fundamental questions without being constrained by traditional academic structures, and working in a research environment with military funding at the time gave me that freedom.

So, those early results didn’t just shape the science—they shaped the direction of my career and the wider field, pointing everyone toward fibre-based QKD as a realistic way forward.

**Q7. In 2007, your team demonstrated a free-space QKD link between Tenerife and La Palma, covering 144** **km. What were the key engineering and atmospheric challenges, and how were they overcome?**

That experiment grew out of a very practical question: if quantum key distribution was ever going to work via satellites, we needed to know how quantum signals would behave when transmitted through real atmosphere. Space-to-ground links inevitably involve atmospheric turbulence, absorption, and pointing challenges.

We began modestly, working with colleagues in Germany to test a 20 km free-space link using lasers and a prepare-and-measure QKD protocol. From there, the idea escalated: why not test a truly long-distance link between the Canary Islands? One of my colleagues pulled out a map showing La Palma and Tenerife—about 144 km apart—and we realised it was a perfect test bed.

A European Space Agency supported project formed to realise this aim, led by Anton Zeilinger’ and Harald Weinfurter joined by my group and other collaborators. The setup benefited enormously from existing infrastructure: on Tenerife, the ESA observatory already had a large telescope and optical laboratory, originally designed for satellite communication tests. That solved part of the engineering problem, but we still had several big challenges.

First, alignment. In QKD, you’re working with one photon per pulse or less, which means you can’t see the signal beam to align it directly. We had to use a much brighter beacon beam for pointing and tracking, carefully filtered out to avoid overwhelming the single-photon detectors, which are extremely sensitive.

Second, turbulence. A telescope with a 40 m focal length is wonderful for astronomy, but for us it magnified tiny atmospheric beam shifts into millimetre-scale motion at the detector plane. To counter this, we built optics to reduce the effective focal length down to about a metre, stabilising the beam footprint on our detectors.

Third, synchronisation. At 144 km, detecting coincidences between photons is like finding needles in a haystack. Background light from stars, scattered light, even headlights from cars in the valleys could trigger false counts. We needed highly precise clock synchronisation to pick out the true photon pairs. That required careful use of GPS timing and clever post-processing.

And finally, there was the sheer practicality of working on mountaintops at night. Our Tenerife site was comfortable inside a telescope dome, but our colleagues on La Palma were operating out of a small hut, freezing cold, taking off their gloves every time they needed to adjust an optical component. Automation and robustness became essential just to keep things running through the night.

Despite these difficulties, we succeeded. The experiment proved that entanglement and QKD could be demonstrated across 144 km of free space—roughly the kind of scale needed for satellite links. For the field, it was a key step in moving quantum communication out of the lab and into real-world environments.

**Q8. Following that breakthrough, your group miniaturised QKD systems for handheld devices and CubeSat payloads. What trade-offs had to be made in terms of performance, size, and cost?**

That work came about partly through necessity. When I moved to Bristol, I no longer had the extensive equipment I’d relied on before. I had to start from scratch, and the question was: what could we build quickly and cheaply to stay in the game?

The idea was bold but simple: could we build a QKD system small enough to fit on a credit card? I pitched this to a European consortium, proposing a system that might cost as little as $10 using off-the-shelf components. The goal was to explore whether quantum security could 1 day reach consumer applications—for example, using a mobile phone to securely upload a PIN to an ATM.

Of course, there were trade-offs. On the transmitter side, cheap LEDs—costing only a few cents—could serve as light sources. That kept costs low and made miniaturisation feasible. But the detectors were the sticking point. Single-photon detectors remained expensive, typically thousands of pounds each. To work around this, we imagined a model where millions of mobile phones could act as transmitters, but the expensive detector units would sit at the receiving end, such as in ATMs.

We also had to make compromises in integration. Full custom chips would have been ideal, but they cost hundreds of thousands of pounds to develop. Instead, we used semi-integrated setups, with discrete components packed as tightly as possible. On the electronics side, we embraced the emerging field of field-programmable gate arrays (FPGAs). These gave us real-time control, clock synchronisation, and the flexibility to adapt the system in software, all at reasonable cost.

The biggest obstacle, though, wasn’t technical—it was commercial. When we approached banks and cryptography experts, we found them cautious. The financial industry is notoriously slow to adopt new technologies, especially in security. They want decades of testing and proof before deploying at scale. So, although the miniaturised handheld QKD systems worked in principle, there wasn’t immediate demand to push them into mass production.

That realisation nudged us in a new direction. If the consumer market wasn’t ready, perhaps satellites were. The same miniaturised systems, with some refinements, could be adapted for CubeSats. In space, compactness, weight, and power efficiency are crucial, so our credit-card-sized transmitters suddenly looked very appealing.

Over the past decade, that’s where our efforts have gone. We’ve built CubeSat payloads carrying full QKD systems in just a few 10 cm cubes of satellite volume. Today, we even have a flight model almost ready for launch, with only a few teething troubles remaining. It’s been a long road, but the journey from lab bench to handheld prototype to CubeSat payload has shown just how flexible and adaptable QKD technology can be.

**Q9. How do you see terrestrial fibre QKD and satellite QKD complementing each other in a global secure network?**

Right now, much of the field is focused on building network demonstrators. The idea is to show that QKD can be integrated into real business models, so we work closely with partners like BT to explore how secure quantum networks might operate in practice.

In metropolitan areas, fibre-based QKD works well. We’ve demonstrated entanglement-based quantum networks on an ITU grid here in Bristol, led by Siddarth Joshi who invented the scheme in the Zeilinger group and brought it to us to develop further. Fibre networks up to now typically use prepare-and-measure schemes, but with our entanglement-based setups we can now link several separate networks through entanglement swapping.

Satellites, on the other hand, are essential for long-haul connections. You can imagine sending short-wavelength photons—say, 800 nm—up to a satellite, while feeding telecom-wavelength photons into a fibre network on the ground. With entanglement swapping, you can effectively link the satellite and the terrestrial network, creating correlations across the globe.

Of course, it’s not yet a solved problem. There are many technical challenges in integrating these pieces, and in countries like the UK, even the weather is a hurdle. One day you have blue skies, the next day heavy rain, which makes satellite links less predictable. If QKD is to become a commercial service, we’ll need strategies to cope with that variability.

But the vision is clear: local fibre networks providing metropolitan coverage, connected via satellite links to extend secure key distribution worldwide. That combination—terrestrial and space—will be the backbone of a future global quantum-secure network.

**Q10. What was your reaction to being awarded the 2023 Micius Quantum Prize?**

To be honest, I was stunned. By then I was semi-retired, spending more time working on a house in Devon that we had recently bought. I’d invested some of my pension and inheritance into the place, and I was out in the garden when the phone rang.

I recognised the number as being from China. I had a suspicion about what it might be, because years earlier, some colleagues had mentioned the idea of nominating me for the prize. I told them not to tell me if they did, since the process is supposed to be anonymous, and over time I had forgotten about it. It felt like buying a lottery ticket years ago and never checking the results.

When I answered, it was Chao-Yang Lu, the chair of the Micius committee. He told me I had won the prize, and I nearly fell over. I walked around the garden in a daze for the rest of the day, hardly able to believe it. Scientists are prone to imposter syndrome—we focus on the work itself and the joy of discovery, not on recognition. So when something like this comes along, it’s overwhelming.

I was bowled over, really. It took me a full day before I could even bring myself to accept it as real. But of course, I’m honoured, and I deeply appreciate the recognition.

**Q11. The same year’s award also honoured Professor Nicolas Gisin. How do you see your respective contributions intersecting?**

Yes, Nicolas and I have collaborated and interacted many times over the years. We first met in the early 1990s, when he too was exploring interferometry-based entanglement experiments. Our groups shared an interest in photon counting and detectors, and we often exchanged ideas and technology.

The development of efficient semiconductor based photon counting detectors was a key underpinning technology that enabled all of the fundamental studies. Having developed Silicon devices in the 1980’s, we moved in the early 1990s to Germanium detectors capable of photon counting at wavelengths used for fibre optic communications. We passed one of these to Nicolas’s group, and they, in turn, helped develop better devices. Their work eventually fed into the creation of ID Quantique, the company that commercialised both detectors and quantum key distribution systems.

Photon-counting detectors really were transformative. Mandel, for example, had been working with photomultipliers that offered only about 2% efficiency. His experiments required hours of data collection. When we switched to silicon photon-counting detectors, the efficiency jumped tenfold (and coincidence counts by a hundredfold), and experiments that once took hours could now be done in minutes. That leap in practicality enabled a wave of new quantum optics and quantum communication experiments.

So, in that sense, Nicolas and I were part of the same story: advancing the tools that made the whole field possible. His group took the next step by commercialising those tools, packaging them into plug-and-play QKD systems that could be deployed outside the lab. I think the Micius committee recognised that our contributions—both in foundational experiments and in translation to applications—underpin much of the present day work in quantum communications technology.

**Q12. How does this recognition reflect the progress of the quantum communication field over the last 30 years?**

When I look back, the progress has been remarkable. In the early 1990s, Charles Bennett and Gilles Brassard had demonstrated the first working QKD system, but it was rather clunky and slow. At the same time, we were working with far better photon-counting detectors and more advanced interferometric techniques. That allowed us to address practical challenges right away—things like switching speeds, transmission distances, and operating at telecom wavelengths.

Our group, working with Paul Townsend, carried out one of the first realistic demonstrations of QKD over 10–20 km of optical fibre. That was important because it moved the field from tabletop feasibility into the realm of practical communication distances. Paul was an expert in fibre optics, while we contributed our expertise in photon counting and interferometry. It was a fruitful collaboration: he showed us how to handle fibres, and we showed him how to count photons.

For me, the evolution of the field reflects exactly that kind of interplay—between theory and experiment, between physics and engineering, between foundational science and practical technology. In just three decades, we’ve gone from slow, fragile demonstrations to robust systems deployed in real networks, and even links via satellites.

So, receiving the Micius Prize isn’t just about my personal contributions. It’s also a symbol of how far the entire field has come—from the first Bell tests with primitive detectors to today’s global vision of a quantum internet.

**Q13. Which technological areas—such as integrated photonics, quantum memories, or detector technologies—do you believe will drive the next phase of development?**

Several areas stand out, and in truth, they all need to advance in parallel if quantum technologies are to fulfil their potential.

First are detectors. Superconducting nanowire detectors have already transformed what we can do. In our lab today, we operate arrays of 40–60 of these detectors, all cooled to cryogenic temperatures detecting single photons with 90% efficiency, and a single array can support a dozen or more experiments at once. That’s an extraordinary leap compared to the days of photomultipliers with just a few percent efficiency. And they’re still improving—expanding sensitivity into the infrared, even out to two or three microns. That opens entirely new application spaces: mid-infrared quantum sensing, environmental monitoring, and free-space communications at wavelengths better suited to the atmosphere. So, detectors will remain a cornerstone of progress.

Next are quantum memories. These are arguably the hardest nut to crack, but they’re absolutely central to the idea of a quantum internet. A memory allows you to capture a qubit, hold onto it while something else happens—perhaps while another entangled photon is being transmitted or a network node comes online—and then release it on demand. That ability to synchronise events across a network is essential for scaling. We’ve seen steady progress in spin–photon interfaces, where a photon’s state is stored in an atomic spin or defect centre and later retrieved. If we can make those devices practical—robust, efficient, and long-lived—they will enable entanglement swapping and truly large-scale networks.

Then there’s integrated photonics, which I think of as the equivalent of the silicon revolution in classical electronics. At the moment, many of our experiments still look like 1980s electronics: large benches, optical tables, and racks of equipment. Photonic integration shrinks that down to chips, with thousands of interferometers, phase shifters, and beam splitters fabricated at once. That makes quantum circuits smaller, cheaper, and vastly more reproducible. It’s the only way to scale from a few dozen qubits to thousands or millions, whether in communication, sensing, or computation. Already, companies and foundries are producing quantum photonic chips with impressive complexity, and the trend is accelerating.

But what excites me most is the interfacing of these technologies. Imagine a network where superconducting qubits in one lab are connected to spin qubits in another, with photons acting as the universal carriers of entanglement between them. Integrated photonics provides the chips to manipulate the photons; superconducting detectors read them out with near-perfect efficiency; and quantum memories synchronise the whole process. The real challenge—and the real opportunity—lies in bringing these elements together.

If I look 10–20 years ahead, I think we’ll see hybrid systems dominate: photons for communication, spins or trapped ions for storage, superconducting, atomic or photonic platforms for processing, all linked by interfaces that allow entanglement to flow seamlessly between them. That, to me, is the fuller vision: not one technology winning out over the others, but a web of complementary advances converging into a scalable, global quantum infrastructure.

**Q14. Alongside technology, what role will international collaboration play in building an intercontinental quantum network?**

International collaboration is absolutely essential. You can’t build a truly global quantum network if every country works in isolation. Unfortunately, politics sometimes gets in the way—leaders appeal to voters by casting other nations as adversaries. That mindset hinders scientific progress.

Science, by contrast, thrives on openness. Our entire field has been built on sharing results: we measure success not only by what we discover, but by how many people read and build upon our work. We stand on the shoulders of those before us—Einstein, Heisenberg, Glauber, Mandel, and many others. Without open publication, each generation would be forced to start from scratch.

Of course, there are limits. Some research inevitably crosses into sensitive territory, like defence applications, where results must remain classified. But the vast majority of quantum communication research benefits from being shared openly, so that others—sometimes brighter than ourselves—can take it further.

If we want an intercontinental quantum network, we’ll need that spirit of collaboration to prevail over politics. Only by working together across borders can we achieve the scale and reliability such a network demands.

**Q15. What advice would you offer to early-career researchers aiming to contribute to both fundamental quantum science and applied systems?**

My advice is to think carefully about what role you want to play. If you want to lead the world in discovery, focus on the fundamentals. That’s where the genuinely new technologies emerge—from ideas that shift our understanding of physics itself. But that’s not the only path. We also need engineers and system builders—people who can take those ideas and translate them into working devices. Both roles are essential.

In my own career, I’ve always gravitated toward the middle ground: taking fundamental concepts, translating them into experiments, and showing they work in practice. But once the experiment is done, it often takes a team of engineers to embed it in hardware, FPGAs, or satellite systems.

So, ask yourself: do you want to be the theorist, the experimentalist, or the engineer? Are you drawn to thought experiments, to lab work, or to building real-world systems? And also be honest about your strengths. If you struggle with maths, don’t force yourself into theory. If you’re impatient with equipment, maybe lab work isn’t for you. Science thrives when people play to their strengths and complement each other.

Above all, keep thinking creatively. Align yourself with strong teams, but don’t be afraid to look outside the box. Many breakthroughs come from people who dared to follow their own path.

**Q16. Looking back over your career—from large-scale optical bench experiments to chip-scale and satellite systems—what has been the most surprising or satisfying development?**

For me, the deepest satisfaction has come from seeing our basic research translate into real-world applications. One example is our work on photon counting, which is now being used in methane-sensing systems to monitor greenhouse gases. It’s gratifying to know that techniques we developed to probe the fundamentals of quantum optics can also address urgent global problems.

Earlier in my career, of course, the greatest thrill was always discovery—observing a new effect, especially when the outcome contradicted our expectations. Those “lightbulb” moments don’t happen every day; sometimes you spend months in the lab before you see a decisive result. But when it comes, it’s exhilarating. These days, it’s usually my students who make the breakthroughs at the bench, but I still share in that joy when we confirm something new.

On a personal level, I get bored easily. That’s why I’ve always sought out the next new thing—something no one has done before. That drive has kept me motivated across decades of research, from tabletop optics to CubeSats.

If there’s a danger in this mindset, it’s that you sometimes miss the commercial opportunities that others later exploit. But that’s fine. Science needs both explorers and entrepreneurs. As long as people can take our ideas and turn them into applications, I’m happy.

So, the most satisfying development is really twofold: the thrill of discovery itself, and the knowledge that some of those discoveries now have tangible value for society. That balance—between curiosity and application—has defined my career.

